# Analysis of tobacco control policies in Nigeria: historical development and application of multi-sectoral action

**DOI:** 10.1186/s12889-018-5831-9

**Published:** 2018-08-15

**Authors:** Oladimeji Oladepo, Mojisola Oluwasanu, Opeyemi Abiona

**Affiliations:** Department of Health Promotion and Education, African Regional Health Education Centre, Faculty of Public Health, College of Medicine, University of Ibadan, Ibadan, Nigeria

**Keywords:** Tobacco, Nigeria, Policy, Non-communicable diseases, Multi sectoral action, “Best buy” interventions

## Abstract

**Background:**

Tobacco use is a major risk factor for non-communicable diseases and policy formulation on tobacco is expected to engrain international guidelines. This paper describes the historical development of tobacco control policies in Nigeria, the use of multi-sectoral action in their formulation and extent to which they align with the World Health Organisation “best buy” interventions.

**Methods:**

We adopted a descriptive case study methodology guided by the Walt and Gilson Policy Analysis Framework. Data collection comprised of document review (*N* = 18) identified through search of government websites and electronic databases with no date restriction and key informant interviews (*N* = 44) with stakeholders in public and private sectors. Data was integrated and analyzed using content analysis. Ethical approval was granted by the University of Ibadan and University College Hospital Ethics Review Committee.

**Results:**

Although the agenda for development of a national tobacco control policy dates back to the 1950s, a comprehensive Framework Convention for Tobacco Control (FCTC) compliant policy was only developed in 2015, 10 years after Nigeria signed the FCTC. Lack of funding and conflict of interest (of protecting citizens from harmful effect of tobacco viz. a viz. the economic gains from the industry) are the major barriers that slowed the policy process. Current tobacco –related policies developed by the Federal Ministry of Health were formulated through strong multi-sectoral engagement and covering all the four WHO “best buy” interventions. Other policies had limited multi-sectoral engagement and “best buy” strategies. The tobacco industry was involved in the development of the Standards for Tobacco Control of 2014 contrary to the long-standing WHO guideline against engagement of the industry in policy formulation.

**Conclusions:**

Nigeria has a comprehensive national policy for tobacco control which was formulated a decade after ratification of the FCTC due to constraints of funding and conflict of interest. Not all the tobacco control policies in Nigeria engrain the principles of multisectorality and best buy strategies in their formulation. There is an urgent need to address these neglected areas that may hamper tobacco control efforts in Nigeria.

## Background

Tobacco use is a major risk factor for non-communicable diseases (NCDs) and has been on the rise in Nigeria.According to the 2012 Global Adult Tobacco Survey, 5.6% (4.7 million) Nigerian adults aged 15 years or older currently used tobacco products (*10.0% of men and 1.1% of women)*. Furthermore, 3.7% (3.1 million) Nigerians currently smoke tobacco and 2.9% (2.4 million) were daily smokers [1]. Moreover, the potential for the number of Nigerian smokers to rapidly increase is significant: the prevalence of smoking experimentation from a nationally representative survey conducted among in-school Nigerian adolescents aged 13–15 years old ranged from 3.6 to 16.2% [2]. More than 60% of males aged 20 to 34 years who ever smoked did so daily before reaching the age of 20 years while 55.3% of current daily tobacco users did so within 30 minutes of waking-up [1]. Exposure to second-hand smoke was reported to be highest among those who visited restaurants; estimated at 29.3% of 6.4 million adults [1]. These data indicate high consumption and may contribute to the incidence and deaths from non-communicable diseases. In 2012, 102,100 new cases of cancer were reported in Nigeria with prostate and liver cancers accounting for over half of the new cases [3]- and these are associated with tobacco use. Furthermore, a total of 792,600 deaths from NCDs were reported during the same period [4]. These may have significant consequences on the country’s socioeconomic development.

As part of the effort to reduce tobacco use, the global tobacco control and NCD prevention efforts were spearheaded by the World Health Organisation, which culminated in the development of the 2005 WHO Framework Convention on Tobacco Control [5]. In 2008, the World Health Assembly endorsed the Action plan for the Global Strategy for the Prevention and Control of Non-Communicable Diseases, proposing actions to address the growing public health burden of NCDs for the period 2008–2013 [6].The Action Plan commits governments to a series of multi-sectoral actions defined as *“actions undertaken by sectors outside the health sector, possibly, but not necessarily, in collaboration with the health sector, on health or health-related outcomes or the determinants of health”* [6]*.* Along with multi-sectoral action, the WHO also proposed policy guidelines underpinned by the “best buy” interventions. A best buy is an intervention that is highly cost-effective cheap, feasible and culturally acceptable to implement in reducing population-level risk and could have significant public health impact, even in resource constrained countries [7]. This policy guideline followed the recognition that the global epidemic of NCDs can be reversed through modest investments in “best buy” interventions which will produce accelerated results in terms of lives saved, diseases prevented and heavy costs avoided [4]. For tobacco control, the WHO “best buy” interventions include tax increases on tobacco products; smoke-free indoor workplaces and public places; bans on tobacco advertising, promotion and sponsorship; mass media campaigns on the harms of tobacco use and second hand smoke and health information and warnings [7].

Nigeria is a member state of the WHO and signatory to the resolutions and conventions adopted at the World Health Assembly (WHA) and other meetings to reduce the harmful use of tobacco, such as the Framework Convention on Tobacco Use Control 2003; WHO African Region Ministerial Consultation on NCDs April 2011; and the United Nations General Assembly September, 2011. It is therefore expected that recommendations from these global commitments would be incorporated in the policy making processes for tobacco control in Nigeria.

Adopting multi-sectoral action in policy formulation is critical to the success of any policy that has underlying factors beyond the health sector. Evidence exists for successful multi-sectoral action in different parts of the world. In the Philippines, a successful multi-sectoral effort between the finance and heath sectors led to the legislation for “sin taxes” [8].Similar achievements have been documented in Thailand [9]. While literature exists on the operationalisation of multi-sectoral action in policy making and potential challenges and opportunities [10–14], it is not known how countries like Nigeria have incorporated this into their tobacco policy making processes.

Drawing from key stakeholder interviews and document review of policies, this paper describes (1) the historical development of tobacco control policies in Nigeria; (2) the extent to which multi sectoral action was applied in the formulation of the tobacco control policies; and (3) the extent to which the WHO “best buy” interventions are included in the tobacco control policies. This article presents findings from a broader study titled “*Analysis for Non-Communicable Disease Prevention Policies in Africa (ANPPA)*”.

## Methods

The study design, recruitment and data collection procedures are detailed in a previously published protocol [15]. This was a case study guided by the Walt and Gilson Framework of Policy Analysis [16] with analyses of historical or contemporary phenomena within real-life contexts [17]. The study assessed policy and practice for all WHO “best buy” interventions and multi-sectoral action in tobacco policy formulation. Two primary sources of data were used: (1) a review of relevant documents related to the policy formulation process and (2) key informant interviews with respondents that either participated or should have participated in the policy development process. According to this study, policy documents include: strategic plans, program plans, guidelines, protocols; parliamentary records or debates; local print media for references to policy changes, meeting minutes, activity reports and drafts of policy statements, internal and external memos, meeting agendas and other communications; academic journal articles; relevant donor or non-governmental organisation and development partner websites for NCD program reports; libraries and internet search engines [15]. Our focus was on the formulation and existence of federal government policy documents and legislation and we did not investigate policy implementation, or policies at the sub-national levels of government.

### Research teams

Our study team comprised of the Principal Investigator, a doctoral student and research manager who attended series of workshops organized by the African Population Health Research Center on non-communicable diseases, health policy, research methods, data analysis, and report writing. The Principal Investigator (OO) has over 3 decades of experience in public health behavioural and policy studies with expertise in qualitative research. The doctoral student (MO) and research manager (OA) have over 10 years of experience in the conduct of public health qualitative and quantitative research studies.

The overall study team developed a toolkit to guide each research team in implementing the study. The tool kit includes description of the study background, the objectives and the procedures for document review, the data collection tool and how to pilot it at country level, ethical considerations, interviewing process, data management procedures and analysis. Data collection took place from June 2014 to September, 2015.

### Document reviews

We searched three online databases (PubMed, Science direct, Google Scholar) and a search engine (Google) using search terms and syntaxes such as (*Nigeria[tiab]) and (Tax or Smoke-free or advertising or promotion or sponsorship or information or warning or access) and (Tobacco) and (policy) and (Multi-sectoral Action)*. The search terms and syntax were modified for each electronic database to retrieve published articles and policy documents written in English Language with no date restrictions. We also contacted relevant key informants and stakeholders to identify other policy documents.

All relevant tobacco-related policies were easily accessed from the websites of government agencies and international partners such as the Tobacco Free Kids. However, minutes of meetings which may have provided more information on the extent of involvement or participation of partners in the policy development processes could not be accessed.

We conducted document reviews to describe the policy context and content, identify existing policies and gaps and understand the policy development processes in respect of best buys and multi-sectoral action.

The review covers specific tobacco control policies in Nigeria and other national and international documents that addressed tobacco control and NCDs prevention. In addition, academic journal articles and relevant reports from donors, non-governmental organisations and development partners were reviewed. MO and OA extracted the key information using an inventory extraction tool with variables such as the document type, author, year, objectives, strategies, and NCD policy element addressed, with emphasis on the best buys, years in which relevant policy changes occurred and the events leading up to those decisions. Each policy document was reviewed and information aligning with these variables was extracted to populate the inventory extraction tool. Some key documents date back to Nigeria’s first tobacco policy such as the 1951 revenue allocation document on licensing and controlling tobacco importation (Section 6 of the Nigeria Order in Council of 1951) [18].

### Key informant interviews

The African Population and Health Research Center and our study team collaboratively developed interview guides during the first methodology workshop. Interview guides were informed by the Walt and Gilson Framework of Policy Analysis [16] and the McQueen analytical framework for inter-sectoral action [19].Questions for tobacco included the context in which the policy was developed, the policy content including the “best buy” interventions, actors involved in the process and the implementation status of each policy. In addition, the extent and processes undertaken to ensure multi-sectoral action and challenges encountered were collected. During field worker training, our team piloted the guide and this was revised based on feedback from the pilot. The final interview guide was used with minor adjustments to fit the Nigerian context.

Interview participants (*N* = 44) were purposively selected based on their expected or actual role in Nigeria’s NCD policy formulation. This was determined using the range of policy actors for NCDs policy formulation as proposed by Meiro- Lorenzo et al., 2011 [20]. These comprise 24 interviewees from various government sectors (*Health, Education, Trade and Investment, Labour, Justice, Information, Finance, Youth and Sports, Women Affairs, Food, Drug Administration and Control and Legislature*); 9 from Research/academic institutions; 6 from NCD associations/ professional bodies, 2 from the hospitality/food industry, 1 each from an International Organisations, NGOs/civil society and religious organisations.

They were identified and selected using a combination of purposive and snowball sampling approach. Senior level policy actors from government and non-government sectors (e.g., professional associations, religious bodies etc.) were recruited and invited to participate in the study via telephone calls or emails. The researchers scheduled an interview appointment and sent an information sheet to those that agreed to participate and subsequently, each interview was conducted for 35 to 50 min.

Only three of the intended policy actors could not be interviewed for various reasons. For instance the WHO Country Representative (international organisation) to Nigeria/designee could not be interviewed due to the exigencies of work associated with the control of the Ebola outbreak in Nigeria while the representative of a government sector stated that the research is of no importance to their ministry and refused to participate in the study. Policy actors especially government officials who participated in the development of current policies were interviewed, however for older policies developed before 1990, this could not be done.

### Data analysis

All interviews were transcribed verbatim to Word files and saved with identification codes on password-protected servers. Data quality checks were ensured throughout data collection and transcription of the interviews. OO, MO and OA listened to the audio recordings and compared to the transcriptions to identify errors/discrepancies in the data. Transcripts were uploaded into the qualitative data management software NVIVO. The African Population and Health Research Center and our study team collaboratively developed a code book based on Walt and Gilson Framework of Policy Analysis [15] to guide coding and ensure consistent classification of themes. MO and OA jointly coded the data using this codebook.

We used thematic analysis [15] to code the documents and transcripts, guided by the key research questions. The extent to which multi-sectoral action was used was measured in two ways: nominal count of the sectors and their level of involvement (i.e. *was voice of stakeholders represented, who funded the process?*) as well as the identification of organisations which have a role in policy formulation but were not involved. In this study, multi-sectoral action was categorized as “high” if ten or more relevant sectors participated in the policy formulation process while five to nine and less than five were categorized as “moderate” or “low” respectively. Results were reported thematically to describe how Nigeria responded to the Framework Convention on Tobacco Control to develop and implement tobacco control policies in accordance with the research questions.

### Ethics

This study was approved by the University of Ibadan/University College Hospital Ethical Review Committee, Nigeria in 2013 before the commencement of data collection (UI/EC/13/0415). Informed and voluntary consent were obtained from all participants as well as the consent to publish the findings. Prior to the interview, the interviewer explained the purpose of the study, risks and benefits to participating, the right to withdraw at any time without penalty, and confidentiality. Participants provided verbal or written documentation of consent to participate. Interviews were conducted at times and venues mutually agreed upon by the research team and the participants. These venues ensured privacy and protection from security risks and the interviews were digitally recorded.

## Results

### Historical context and process of tobacco control policies in Nigeria

Data in this section reflects findings from the document review and key informant interviews. The development of tobacco control policies and legislations in Nigeria have been an eventful pathway. As depicted in Table 1, the first attempt by the Nigeria government on tobacco control appeared in a 1951 revenue allocation document on licensing and controlling tobacco importation - Section 6 of the Nigeria Order in Council of 1951 [18, 21].This policy document focused largely on the regulation of tobacco trade specifically the licensing, importation of tobacco and payment of duties [21]. The first major attempt to regulate tobacco use for health-related reasons occurred four decades later starting with the formulation of the Tobacco Smoking (Control) Decree 20, 1990 [22] by the military government. This was converted to an Act (“Tobacco Control Act 1990 CAP.T16”) when Nigeria transited to democratic rule in year 2000 [23].While the content of both documents remained unchanged, the use of “Decree” by the military and “Act” by democratic government was due to different terminologies of governance tools. This Act regulated tobacco control for over two decades. However, the policy was weak and poorly implemented as shown in a 2008 WHO report [18, 24]. This report and Nigeria’s ratification of the Framework Convention on Tobacco Control (FCTC) triggered the tobacco control community to mount significant pressure in 2009. This resulted in the development of a FCTC-compliant comprehensive tobacco control bill called the “National Tobacco Control Bill 2009”and entitled “A Bill for an Act to Repeal the Tobacco (Control) Act 1990 Cap T16 Laws of the Federation and to Enact the National Tobacco Control Bill 2009”. This bill was aimed at providing regulation for the control of production, manufacture, sale, advertising, promotion and sponsorship of tobacco or tobacco products in Nigeria. This legislative Bill, expected to be passed through and approved by a body of legislators called the “House of Assembly” and “Senate” at the national level had a tortuous process. The 2009 National Tobacco Bill was sponsored by the Lagos East senator and deputy minority leader, Dr. Olorunnibe Mamora and received its official second reading in February 2009.The British American Tobacco, Nigeria (BATN) according to tobacco control advocates and interviewees actively sought to halt its passage and prevent the bill from advancing to the committee stage. However, the vigorous efforts of civil society organisations countered those actions. The bill was then considered in a formal public hearing by the Senate Health Committee in July 2009 and received a major boost from Professor Babatunde Osotimehin, the former Minister of Health, and Senator Jibrin Aminu, a former minister, ambassador, two-time senator and chairman of the Senate Committee on Foreign Affairs, who publicly spoke out in support of the proposed legislation [23]. The bill also received strong support from many domestic and international civil society groups. Three Nigerian NGOs (Environmental Rights Action/Friends of the Earth, Nigeria; the Nigerian Tobacco Control Alliance; and the Coalition Against Tobacco) also contributed support. This enormous support notwithstanding, the bill again met with strong opposition from the BATN, which used tactics to stall its passage. For instance, the BATN sponsored a full page advert in The Guardian to undermine the anti-tobacco NGOs by informing the public of the “aggressive propaganda against the Tobacco Industry” and stating that they must be part of the solution based on their strong efforts at controlling tobacco use contrary to the WHO FCTC [25].

**Table 1:** Overview of Tobacco control policies in Nigeria

Year/Source	Policy	Content
1951, Section 6 of the Nigeria (Revenue allocation) Order in-Council	Revenue allocation document	licensing and controlling tobacco importation
1990, Tobacco Smoking (Control) Decree 20	Tobacco Smoking (Control) Decree 20, 1990	banned smoking in specified public places, and it required warning messages on every tobacco advertisement and sponsorship.
1990, Tobacco (Control) Act 1990 CAP.T16) changed after Nigeria transitioned to democratic rule in year 2000	Tobacco (Control) Act 1990 CAP.T16)	same as for 1990.
2009, National Tobacco Control Bill	National Tobacco Control Bill 2009 Passed in 2011 but president refused to sign in 2013.	regulation or control of production, manufacture, sale, advertising, promotion and sponsorship of tobacco or tobacco products in Nigeria
2014, Standard Organisation of Nigeria, Tobacco and Tobacco Products - Specifications for Cigarettes	2014 Standard for Tobacco and Tobacco Products - Specifications for Cigarettes	compliant specifications on how the packaging for cigarettes (i.e. cartons, rolls and individual packets) should be marked with health warnings on the dangers of tobacco use and prohibits the use of flavouring substances with potential to initiate or appeal to children
2015, National Tobacco Control Act	The National Tobacco Control Act 2015	Tobacco Control Bill 2009 was modified to include establishment of Tobacco control committee and tobacco control unit; Tobacco control funding; prohibition of smoking in public places; prohibition on tobacco emissions disclosure; tobacco products packaging and labeling; enforcements and roles of responsible Organisation; education, communication and public awareness and miscellaneous including price and tax measures
Developed in 2013 but reviewed in 2015.	Nigerian National Policy and Strategic Plan of Action on Non-Communicable Diseases	protecting people from tobacco smoke in public places and work places, warning people about the dangers of tobacco, enforcing bans on advertising, promotion and sponsorship, and raising tobacco tax and prices.

The Bill was eventually passed by the Houses of the National Assembly and Senate and sent to the President for final assent in 2013 but the presidency failed to sign the bill due to further pressure from the tobacco industry and the objection by some government ministries which felt they should lead some aspects of the tobacco control measures and government bureaucracy. With the failure of the bill due to lack of presidential assent, another version was developed by the Federal Ministry of Health and passed as an Executive Bill to the Federal Executive Council and the Senate for approval. Eventually the Bill was finally approved by the Senate in the midst of supportive pressure from the civil society and formidable counter resistance from the tobacco industry. The Bill was forwarded to President- Goodluck Jonathan who signed the Executive Bill into law on the 27th of May, 2015 (shortly before his exit) as the “National Tobacco Control Act 2015”. This Bill is a comprehensive, FCTC-complaint legal instrument which addresses all the Tobacco “best buy” interventions as well as other measures relating to the reduction of the demand and supply for tobacco and related matters [26]. The specific policy elements addressed are shown in Table 1. The critical factors which aided the passage of the Act were the pressure from the civil society and other stakeholders, and the president’s decision of leaving a mark of achievement at the end his term as president.

Review results further show that two other policies: the Nigerian National Policy and Strategic Plan of Action on Non-Communicable Diseases developed by the Federal Ministry of Health in 2013 and reviewed in 2015 [27] and the “2014 Standard for Tobacco and Tobacco products-specifications for cigarettes,” [28] developed by the Standards Organisation of Nigeria (*a government regulatory agency for manufactured product*) coherently align with the National Tobacco Control Act 2015 as both address the “best buy” interventions using the provisions of the WHO Framework Convention on Tobacco Control. However, a major observation was the involvement of the tobacco industry in the formulation of the “2014 Standard for Tobacco and Tobacco Products” contrary to the recommendations of the Framework Convention on Tobacco Control. The Standard Organisation of Nigeria justified the inclusion on the basis of the expectation that policy is expected to guide the manufacturing activities of the tobacco companies.

### WHO “best buy” interventions included in the tobacco control policies

The Tobacco policies address some of the FCTC recommendation and the WHO best buy intervention. These are summarized in Table 2.

**Table 2:** Tobacco policies, the “best buy” interventions addressed and year of development

Recommended WHO Best-Buy interventions	Policy Documents Reviewed	WHO “Best-Buy” interventions addressed in the policy document
*Best buy 1*:Tax increases	National Policy and Strategic Plan of Action on Non-Communicable Diseases 2013	All best buy interventions
*Best buy 2:*Smoke-free indoor workplaces and public places	Tobacco (Control) Act 1990 CAP.T16	Best buy 2,3 and4
*Best buy 3*: Bans on tobacco advertising, promotion, and sponsorship	National Tobacco Control Act 2015	All best buy interventions
*Best buy 4*: Health information and warnings	Standard for Tobacco and Tobacco products-specifications for cigarette 2014	Best buy 4
*Best buy 5:* Mass media campaigns on the harms of tobacco use and second hand smoke		

The National Tobacco Control Act 2015 [26] and the 2013 Nigerian National Policy and 2015 Strategic Plan of Action on Non-Communicable Diseases [27] address all the “best buy” interventions using the provisions of the WHO Framework Convention on Tobacco Control. For example, the Part III, Sub-section 9 of the National Tobacco Control Act 2015 addresses the prohibition of smoking in public places; Part V, Sub-section 12 outlines actions for the prohibition of tobacco advertising, promotion and sponsorship; Part VIII, Sub-section 20 specifies that tobacco products package shall contain health warnings in writing and graphics which shall not be less than 50% of the total surface area of the package,, Part XII, Sub-section 38 states that there will be active promotion and strengthening of public awareness on the health consequences, addictive-nature and threats posed by tobacco use through a comprehensive nationwide education, information and communication campaign organized through ministries and agencies of government in collaboration with civil society organisations and Part XII, Sub-section 43 addresses tobacco taxation and price measures. Aside from the “best buy” interventions, other tobacco supply and demand measures proposed include the establishment of the national tobacco control committee and the tobacco control unit; establishment of a tobacco control fund; regulation of tobacco product sales; regulation of tobacco products content and emissions disclosure; licensing of tobacco dealers; enforcement activities; training for the general populace; and public awareness campaigns. Though there are strong points in the act, some gaps still exist. The requirements for the health warning are textual messages, written only in English language without the use of graphics or pictorials which have the ability to convey more information to most Nigerians who are non-literate. In the case of the 2013 Nigerian National Policy and 2015 Strategic Plan of Action on Non-Communicable Diseases, the content includes specific proposed interventions: (1) Protecting people from tobacco smoke in public places and work places, (2) Warning people about the dangers of tobacco, (3) Enforcing bans on advertising, promotion and sponsorship, and (4) Implement public awareness programmes on tobacco and other NCDs risk factors and (5) Raising tobacco tax and prices.

However, the National Tobacco Control Act 1990 [22] and the 2014 Standard for Tobacco and Tobacco products-specifications for cigarette do not address all the FCTC recommendations and the WHO best buy interventions.

Limiting tobacco smoking in public places, restriction on tobacco smoking advertisement, tobacco warning labels and information on packages were included in the National Tobacco Control Act 1990. However; taxation and price measures are not addressed and so are other tobacco supply and demand measures. Besides, the penalties for violations are generally weak [23] and no longer relevant- twenty-six years after its ratification. Similarly, observations were made in respect of the 2014 Standard for Tobacco and Tobacco products in which only one of the “best buy” interventions on adding health information and warnings to cigarette packaging is in line with the FCTC but not others.

### Multi-sectoral approaches in the formulation of the tobacco control policies

Findings presented below are based on a triangulation of data from document review and key informant interviews. Multi-sectoral action was used in varying degree in tobacco control policy formulation. These are summarized in Table 3.

**Table 3:** Scoring for multi-sectoral action in tobacco policy formulation

Sectors	1990 Tobacco Act and Decree	National Tobacco Control Act 2015	Standard for Tobacco and Tobacco products-specifications for cigarette 2014	National Policy 2013 and Strategic Plan of Action on Non-Communicable Diseases 2015	
				Initial Formulation in 2013	After the review facilitated by the WHO in 2015
Federal Government Ministries/Departments /Agencies					
Health	®	**√**	**×**	**√**	**√**
Education	®	**√**	**×**	**×**	**√**
Agriculture	®	**√**	**×**	**×**	**√**
Environment	®	**√**	**×**	**×**	**×**
Justice	®	**√**	**×**	**×**	**×**
Finance	®	**√**	**×**	**×**	**√**
Sports	®	**×**	**×**	**×**	**×**
Urban and regional planning	®	**×**	**×**	**×**	**×**
Transport	®	**×**	**×**	**√**	**√**
Women Affairs		**√**	**×**	**×**	**√**
Trade	®	**√**	**×**	**×**	**√**
Legislature	®	**√**	**×**	**×**	**√**
Regulatory agencies	®	**√**	**√**	**×**	**√**
Research and Academic	®	**√**	**√**	**√**	**√**
Law enforcement	®	**√**	**×**	**×**	**×**
Information	®	**√**	**×**	**×**	**√**
Private and Non-government sectors					
Professional associations	®	**√**	**×**	**√**	**√**
Civil Society Organisations/	®	**√**	**×**	**√**	**√**
Religious Organisations	®	**√**	**×**	**√**	**√**
Tobacco Industry	®	**×**	**√**	**×**	**×**
Other Industries/Private sector	®	**×**	**×**	**×**	**√**
Total Number of sectors involved		16	3	6	15
MSA Rating		High	Low	Moderate	High

®No information

**√**Involved

**×** Not involved

Applying the rating (high, moderate and low) on the use of multi-sectoral action in the tobacco policy formulation (described in the methodology section), overall findings show a range of low to high depending on the policy reviewed. The National Tobacco Control Act 2015 [26] had all the rich blend of relevant sectors involved in its formulation and was adjudged “high” for multi-sectoral action (see Table 3). The 2013 National Policy and 2015 Strategic Plan of Action on Non-Communicable Diseases [27] initially indicates a moderate level of multi-sectoral action as several relevant sectors pertinent to the implementation of the policies were not involved in the formulation process (see Table 3). However, this gap was partially addressed based on the recommendation of the World Health Organisation Nigeria, as the committee that developed the policy document was reconstituted with a broader representation and the rating was subsequently adjudged high. The 2014 Standard for Tobacco and Tobacco products-specifications for cigarette [28] does not have all the relevant sectors and was adjudged “low” in multi-sectoral engagement. The 1990 National Tobacco Decree and Act were not rated in the absence of information on stakeholder involvement (see Table 3).

### Facilitators to multi-sectoral action

The summary below largely reflects findings from the key informant interviews. Findings revealed various facilitators which promoted multi-sectoral level involvement in the formulation of the 2015 Tobacco Act, and the 2013 National Policy and 2015 Strategic Plan of Action on Non-Communicable Diseases which are fairly recent policy documents. One of these, was the understanding among members of the tobacco committee that, the Federal Ministry of Health cannot solely implement actions proposed for tobacco control without the involvement of all relevant line ministry/organisation as illustrated in the quotes below.

“*The act cannot be operationalised by the Ministry of Health alone; it cuts across all the players. The section that Agriculture will enforce, they should know that they will enforce in a particular section……. Customs should know they are collecting adequate revenue for government. So, everyone, every player has a stake.”* (Federal Ministry of Health Official).

*“Well involving all these sectors would ensure that [the activities and strategies] are implemented and would also ensure that there is a national frame work into which the activities can fit into*” [Respondent 1 from Academic and Medical Sector,].

Another facilitator which enhanced multi-sectoral action was the perception that the involvement of all relevant sectors will produce a quality tobacco legislation which will address their interests.

*“[If you bring in relevant sectors,]* … *you have a better document, a tested document that will appeal to everyone, ……… so you have a better law in place when all the sectors are involved*” (Member, House of Representatives).

The funding support for the conduct of the multiple consultative meetings for the drafting and development of the tobacco bill also engendered multi-sectoral action making it possible to invite and support the participation of more stakeholders at the various meetings conducted as reflected below:

*“We had support from WHO and also the Campaign for Tobacco Free Kids who funded the meetings.” (Federal Ministry of Health Official).*

In addition, some sectors funded the participation of their representatives at these meetings thus reducing the financial burden for the Federal Ministry of Health and invariably aiding multi-sectoral action.

“*Yes, we [Federal Ministry of Health] had challenges of funding but we tried our best to circumvent such and many groups were willing to fund themselves and support themselves without collecting a dime” …... (Federal Ministry of Health Official).*

The sharing of ideas from diverse sources with enhanced output, increased sense of joint ownership through bringing people together, and potential sustainability. Were identified as facilitating factors for multi-sectoral action as illustrated in the quotes below:

“*You have diverse perspectives being brought together and there are things that will be hidden from agency A and because agency B is exposed to different view, or different perspective or different area, they are able to cover it, so you have a wider reach in terms of policy document and even in terms of implementation*” [Official of Government Regulatory Agency].

“*One of the benefits is ownership because we are bringing people along; everybody has a sense of belonging in the policy implementation. It is more successful, it faster and people see it as their policy, their programme because there is series of activities, and those activities are their activities … so, there is sustainability”*. [Official of Federal Ministry of Health]

### Barriers to multi-sectoral action

The summary below largely reflects findings from the key informant interviews with a focus on the 2015 Tobacco Act, and the 2013 National Policy and 2015 Strategic Plan of Action on Non-Communicable Diseases. Participants stated various barriers to bringing different sectors to work together including contentions with regards to the appropriate ministry or government agency to take the leadership position and drive the policy formulation process, resource allocation, joint coordination and funding of activities.

“*Let me say that tobacco policy brought together a lot of stakeholders majorly but … at the ministry levels, it created rivalry. In many ministries, [felt they] should be in charge of certain aspects of the tobacco control bill and that created a lot of setback and also was a major factor in the 2009 tobacco bill not being accepted by Mr. President because of objection by certain ministries. One of the challenges was that who [which government ministry or agency] should be at the driver’s seat*” [Respondent 1 from Academic/ Medical Sector].

In addition, participants expressed resource allocation and funding including lack of clarity about mandates and mechanism for obtaining resources as a challenge which hindered multi-sectoral action as expressed below:

*For instance, using the XXX agency as an example now, the people told me we should work together with ABC [sector/ministry], but how do you work with ABC when your resources come differently? Any activity that we undertake, we have to provide the resources to do that, like lunch, money and also, if you have a multi-organisational or multi-agencies working arrangement. You are now saddled with the logistic arrangement because you have to agree on who is to do one thing and who is to bring what before you can do it [implement activities].Whereas, once [our agency is] determined that we want to do something, we take off and we do it.* [Official of the Food regulatory agency]

*“I think the reason [for not involving all relevant sector] is possibly funding; you know to get a policy done is a lot of ceremony, getting a lot of stakeholders, various jurisdiction and various multi stakeholders. So, for now, we have articulated the entire policy cycle, how to start it and conclude it, but it has not yet started, it takes a minimum of possibly 3, 4 or 5 meetings with different stakeholders, various organisations, various interest groups, and then we have challenges due to potential conflicts of interest, as we are not supposed to involve the industries in the policy formulation.”* [Respondent 2 from Academic and Medical Sector].

*“Yes, we had challenges of funding [for the meetings] but we try our best to circumvent such ………that is in addition to the support from WHO and Campaign for Tobacco Free Kids” *[Official of the Federal Ministry of Health].

Other challenges stated by participants include resources and the conflict of interest with regards to the modes of operation of the different sectors and their relationship with the tobacco industry.

*…. The Standard Organisation of Nigeria believes that the tobacco industries are stakeholders and therefore…. the tobacco industry must be present. Whereas the health ministry believes that it is a health issue and the industry has stood in the way of progress in the passage of the tobacco control bill. … The Standards Organisation of Nigeria believes also that they should be in charge of regulation and that they are already lost within the tobacco act [*i.e. *no clear mandates with regards to their core functions*] *…..and there is need for a separate law [for regulation].*[Respondent 2 from Academic and Medical Sector].

These findings highlight factors promoting and hindering the use of multi- sectoral action for the formulation and implementation of tobacco policies.

### Extent of implementation of tobacco control policies in Nigeria

Prior to the enactment of the 2015 Tobacco control Act, the Nigerian Government implemented some activities/interventions based on the National Tobacco Control Act 1990. Specifically in this policy document, there was a ban on *tobacco advertising, promotion, and sponsorship* on radio, television and print advertising however, this was not enforced [23]. This supports findings from the Global Adult Tobacco Survey in Nigeria which showed that 21.5% of adults aged 15 years and over noticed any advertisement or promotion of cigarette marketing during the last 30 days [1]. Furthermore, the 2015 WHO Report on the Global Tobacco Epidemics lists Nigeria as one of the countries with a complete absence of ban, or ban that does not cover national television, radio and print media [29]. Unfortunately, the 2015 Tobacco Act failed to completely ban advertising, promotion, and sponsorship at the point of sale, in domestic print media, and other media such as pamphlets and flyers and stipulates that consenting adults can have advertisement and promotion extended to them [30] and this is a significant gap for effective tobacco control in Nigeria.

With regards to mass media campaigns on the harms of tobacco use and second hand smoke, the WHO Report on the Global Tobacco Epidemics has no information [29] but anecdotal information suggests minimal interventions have been implemented in this regard.

Although the Nigeria’s Tobacco Act 1990 and 2015 prohibit smoking in public places, the extent of enforcement and implementation remained low and Nigeria is listed as one of the countries with only three to five public places completely smoke-free [29]. This aligns with findings from the Nigeria Youth Tobacco Survey (2008) which reported a high proportion of young people exposed to secondhand smoke in public places ranging from 35% in Ibadan to 46.9% in Cross Rivers and 55.8% in Kano [2]. The 2012 GATS also found that the percentage of adults aged 15 and older exposed to tobacco smoke in government buildings, public transportation, restaurants, and bars in the 30 days preceding data collection were 3.5, 6.9, 7.9, and 7.2 respectively [1].

The 2014 Standard for Tobacco and Tobacco Products – Specifications for Cigarettes mandate *that health information and warning occupy 50%* percentage of the principal display area on the front and back panels of each cigarette pack [28]. However, compliance with this directive is inconsistent [31]. Furthermore, Nigeria relies only on the mandatory text-only warning and graphics are not placed on the tobacco packs [29, 32].

Tobacco taxation was implemented to an extent. For instance, in 2008, the share of tobacco-specific tax on widely consumed of cigarettes in Nigeria was 28%. However, this is abysmally low compared with other African countries, such as Ghana or Seychelles, which had a total tax of 55 and 79%, respectively [24]. The 2015 WHO report indicated a further decline in the total tax for Nigeria (20.63%) although it is recommended that tobacco excise taxes be set above 70% of the retail price of the product to increase prices and reduce consumption [29].

The 2015 National Tobacco Control Act enacted in May 2015 is FCTC-compliant, but implementation has not commenced despite an inaugurated committee in July 2016 to guide it. The national NCDs strategic plan and policy was published online in a draft form, but not disseminated to states for adoption and ratification of the proposed actions. Implementation is yet to commence.

## Discussion

Findings from this study revealed a ten-year gap between Nigeria’s ratification of the Framework Convention on Tobacco Control in 2005 and the formulation of a comprehensive tobacco bill and its passage into an Act in 2015. This was largely attributed to the strong opposition and lobbying of the tobacco industry, the compromising stance of the government of Nigeria which considered economic gains over the harms of tobacco products on population health and the bureaucratic legislative processes. It can be argued that the strong lobbying of the tobacco industry most likely fueled the government economic position and the bureaucratic process. This argument is apt in light of the report that in 2001, the Nigerian government signed a memorandum of understanding with British American Tobacco Nigeria (BATN) to build potential for regional export and significantly increase the quantity and quality of locally grown tobacco [33]. Furthermore, Drope 2011; Agaku et al. 2012 and Premium Newspaper 2013 [23, 34, 35] attributed the presidency’s failure to give assent to the bill in 2013 to the counter-lobbying of the tobacco industry. Similar findings of strong opposition and lobbying against the Tobacco Products Control Amendment Bill have been reported in South Africa, and other low and middle income countries [36, 37]. These observations probably justify WHO’s position on the exclusion of the tobacco industries in Tobacco policy formulation. According to the WHO Framework Convention on Tobacco Control, countries should not involve the tobacco industry in the development of tobacco policy in order to forestall their undue interference in undermining its content. Thus, the justification of the Standard Organisation of Nigeria, on the involvement of the tobacco industry, premised on the need to involve them in the development of manufacturing guidelines is contrary to the requirements of the development of the tobacco bill as proposed by WHO and was an issue in the multi-sectoral action process.

Findings also showed the effect of the Framework Convention on Tobacco Control in accelerating conformity of government policies to international agreed standards. It was noted that prior to the ratification of the Framework Convention on Tobacco Control, some of the tobacco policies did not comprehensively address the “best buy” interventions. However later policies covered all “best buy” interventions. For instance, from the findings of this study, two major policies formulated after the FCTC ratification in Nigeria fully complied with the principles of multi-sectoral action and the five recommended WHO “best buy” interventions [26, 27] while the earlier policies addressed the “best buys” partially.

It should however be noted that the operationalisation of multi-sectoral action is fraught with its own potential challenges especially when different sectors have conflicting objectives over values and diverging interests (economic or otherwise) [13]. As reflected in the findings of this study, the Non-Communicable Diseases Division of the Federal Ministry of Health spearheaded the sectors involved in the formulation of the tobacco policies. This might be a reflection of the traditional notion of this ministry being the only entity for population health guidance and programmes, discounting the reality that other sectors outside the health are as important, if not more important in providing strategic directions for dealing with the burden of tobacco use. It further underscores the importance of achieving consensus across all partners to reach a “shared vision” and a common ground where each institutional vision lies within [38].

The findings in this study further provide an understanding on how the formulation of tobacco policies is shaped by a myriad of factors, specifically, how the constellation of external and internal factors shape policy formulation to engrain global principles such as multi-sectoral action and “best buy” interventions. While government participation in high level global meetings is a demonstration of political commitment, most of the barrier factors are traceable to government inaction especially inadequate funding from government to support the process of policy formulation and over dependency on donor Organisations reflecting a lack of sustained leadership and strong political support [39]. The use of the Walt and Gilson Policy Analysis Triangle for this study provided a rich descriptive analysis and narrative of the development of tobacco control policies in Nigeria. This is particularly useful in highlighting how policy issues emerged, how it was developed and the current status. To enhance understanding of the policy dynamics, it is useful to conduct an explanatory analysis using one or more policy process theoretical frameworks such as the Multiple Streams or Advocacy Coalition Framework and this is recommended for further tobacco policy research study [40]. A systematic assessment of the extent of implementation and enforcement of tobacco control policies in Nigeria falls outside the remit of this study and further research in this direction is recommended.

## **Conclusio**n

Overall, this policy analysis provides useful assessment of the national government’s response to the adoption of global declarations and guidelines on tobacco control policy formulation and highlights areas needing attention. The findings of this study are pertinent especially in view of the resolve of the current government administration to strengthen tobacco control in Nigeria. Hence, findings could guide government and non-governmental organisations involved in policy making by drawing attention to issues needing major attention.

### Recommendations

In line with objective 2 of the 2008–2013 Action Plan for the Global Strategy for the Prevention and Control of Non-Communicable Diseases, Nigeria should develop the “best buy” interventions implementation plan using a multi-sectoral action committee supported with adequate resources. The committee should review and update the tobacco control act and other related policies to ensure it has stronger actions.

Policy bureaucrats especially technocrats at the government ministries such as Directors and programme managers, should be urgently equipped with multi-sectoral action and “best buy” interventions knowledge as well as skills on the use of dialogue, consensus and group dynamics to achieve the common goal in NCD policy development [8].

To overcome funding challenges for policy formulation and implementation activities on a sustainable basis, there is a need to ensure that tobacco excise taxes are set above 70% of the retail price of the tobacco product to significantly increase the prices and reduce consumption. In addition, a proportion of the tobacco taxes should be allocated for funding tobacco and NCDs prevention and control efforts.

### Strengths and limitations

We experienced some limitations in the conduct of this study. Firstly, we could not reach all the policy makers that participated in the formulation of the tobacco policies due to the Ebola outbreak. Some of the actors concerned left the country and could not be reached and in some instances, this led to rescheduling some of the interviews. However, we were able to obtain stakeholders from both the public and private sector which presents opportunity for a good mix of information collected. Secondly, the research team could not access minutes of meetings which can help provide information on the extent of involvement and participation of actors in the policy processes coupled with the recall bias among key informants in describing some of these past events and efforts. Thirdly, there is a limit to using a case study approach as the results obtained in this study is not generalisable to countries due to Nigeria’s unique culture and political milieu that shapes the context and content of the policies.

## Abbreviations

ANPPA: Analysis of non-communicable disease prevention policies in Africa
BATN: British American Tobacco, Nigeria
FCTC: Framework convention on tobacco control
MSA: Multi-sectoral approach
NCDs: Non-communicable diseases
WHA: World health assembly
WHO: World health organisation
